# The experience of online cardiac arrest video use for education and research: A qualitative interview study completed in partnership with survivors and co-survivors

**DOI:** 10.1016/j.resplu.2023.100394

**Published:** 2023-05-13

**Authors:** Matthew J Douma, Christopher T Picard, Peter G. Brindley, Jennifer Gibson

**Affiliations:** a Nursing, Midwifery and Health Systems, University College Dublin, Ireland; b Department of Critical Care Medicine, University of Alberta, Edmonton, Alberta, Canada; c Faculty of Nursing, University of Alberta Edmonton, Alberta, Canada; d Department of Critical Care Medicine, University of Alberta, Edmonton, Alberta, Canada; e Providence Health Care, Canada; f University of British Columbia Vancouver, British Columbia, Canada

**Keywords:** Cardiac arrest, Resuscitation, Video, Internet, Cardiopulmonary resuscitation, Ethics

## Abstract

**Background:**

Swift recognition of cardiac arrest is required for survival, however failure to recognize (and delayed response) is common. Studying online cardiac arrest videos may aid recognition, however the ethical implications of this are unknown. We examined their use from the perspective of persons with lived experience of cardiac arrest, seeking to understand the experience of having one’s cardiac arrest recorded and available online.

**Methods:**

We gathered qualitative data using focused interviews of persons affected by cardiac arrest. Inductive thematic analysis was performed, as well as a deductive ethical analysis. Co-researcher survivors and co-survivors were involved in all stages of this project.

**Findings:**

We identified themes of ‘shock, hurt and helplessness’ and ‘surreality and reality’ to describe the experience of having one’s (or a family member’s) cardiac arrest captured and distributed online. Participants provided guidance on the use of online videos for education and research, emphasising beneficence, autonomy, non-maleficence, and justice.

**Conclusions:**

Finding one’s own, or a family member’s cardiac arrest video online is shocking and potentially harmful for families. If ethical principles are followed however, there may be acceptable procedures for the use of online videos of cardiac arrest for education or research purposes. The careful use of online videos of cardiac arrest for education and research may help improve recognition and response, though additional research is required to confirm or refute this claim.

## Introduction

Video recording of everyday life is increasing. As of 2021 there were over seven hundred and seventy million government-controlled closed circuit television (CCTV) cameras, most of which are located in China.^1^ Other notable high surveillance cities include London (73 cameras/1000 persons), Hyderabad (37 cameras/1000 persons), Delhi (34 cameras/1000 persons), Singapore (18 cameras/1000persons), Baghdad and Moscow (16 cameras/1000persons).^1^ In 2020, there were over 3 billion smartphones with recording capability worldwide^2^ and over 500 hours of video uploaded to YouTube each minute.^3^ Medical research increasingly uses videos to study epilepsy^4^, ^5^ and infantile spasms.^6^ Resuscitation scientists have studied sudden cardiac arrest (CA) during televised sports,^7^, ^8^ body cameras during pre-hospital CA^9^ and bystander YouTube videos of cardiopulmonary resuscitation (CPR) performance.^10^

CA remains a leading cause of death, despite advances in resuscitation science. The first link in the chain of survival is successful recognition by lay persons, health care workers, or emergency call takers. Early recognition is crucial to initiate time-sensitive rescue procedures.^11^, ^12^ Recognition of CA and activation of emergency response procedures can be hampered by a failure to appreciate the victim’s inability to respond, the absence of breathing, and further complicated by gasping or agonal breathing and seizure,^13^, ^14^ vomiting or hemoptysis.^14^ Furthermore, there may also be critical sex-related differences in recognition and response.^15^, ^16^

Videos of cardiac arrest are being uploaded to online video-sharing platforms every day, often without the consent of the persons recorded. Canadian research ethics guidance advises that videos in the public domain may be utilized for training and research without additional ethical considerations, such as obtaining permission from the persons in the video or notifying them that the video is being used to offer a mechanism to opt-out.^17^ Some early and incomplete efforts have been made to study videos of sudden cardiac arrest,^18^, ^19^ but were paused to explore the impact of the videos’ proliferation, sharing and use for education and scientific purposes from the perspective of survivors and families. There is an emerging body of knowledge that suggests there are important ethical (consent and privacy), legal (ownership and harm) and mental health implications (trauma, anxiety, shame) resulting from the sharing of online videos.^20^, ^21^, ^22^ In this current study, we partnered with CA survivors and family members of survivors and non-survivors alike to understand i) the experience of having a CA captured on video and shared online (actual or hypothetical); and ii) whether online videos of CA should be approached, studied, or shared by educators and researchers; and iii) if yes, how?

## Methods

### Data collection

This work is part of a larger research programme exploring the care needs of survivors and families post-CA^12^, ^13^, ^14^ (https://osf.io/fxp5g/).^15^ Here, we report qualitative results from semi-structured interviews conducted with survivors of CA and family members of CA survivors and non-survivors, some of whose CA were captured on video and uploaded to the internet from surveillance cameras or bystander recorded video without their consent. Individual interviews were chosen over focus group interviews to explore personal experiences more thoroughly rather than group norms and social interactions.^16^ Furthermore, we wished to avoid unpredictable discussions/interactions and allow participants to revoke their consent.

Twenty-four participants were recruited using snowball sampling. Interviews took place between April 2020 and July 2021 via Zoom^TM^ (San Jose, California) using previously validated methods for virtual qualitative research.^17^ Interviews were performed by MJD a doctoral student with training and experience in qualitative data collection methods. The audio was recorded and stored for transcription. An interview guide was developed a priori and refined during data collection.

### Data analysis

Data analysis was performed iteratively and alongside data collection until sufficient data was obtained to answer the research questions and recruitment was ceased.^18^ Thematic analysis was used to identify themes.^19^ We repeatedly listened to the audio recordings and read the transcripts to derive codes, using both deductive reasoning (using our interview guide) and inductive reasoning (using the data obtained).^20^ Only data relevant to the research aims were coded^21^ using Taguette software (New York, USA).^22^.

Data analysis was carried out primarily by MJD and PGB with analytical input from three co-researchers with experience of CA, who also reviewed a subset of transcripts.^23^ During analysis, codes with common elements were grouped under subthemes and categorized under core themes.^18^ The co-researchers examined data coherence within each theme and the appropriateness and accuracy of each theme. Study authors independently reviewed the themes and agreed upon a final interpretation before sending the final themes and descriptions to all 24 participants with an invitation for input. No subsequent changes were made to the coding or themes following participant verification.

### Patient and public involvement

Of the 24 persons interviewed, two survivors and one co-survivor were involved in the study’s design and processes as co-researchers. Twelve participants reviewed a summary of the study findings when we shared our data and analysis to obtain feedback on accuracy and clarity. No participants dropped out prematurely nor did they express criticism or request clarification about the findings. Co-researchers received monetary honoraria for their time as well as reimbursement for expenses.

### Reflexivity

Our writing team are healthcare professionals with expertise in cardiovascular care, ethics and resuscitation. We believe that handling these videos requires i) an approach guided by affected survivors and family members, and ii) and adherence to professional and ethical obligations. Trustworthiness in qualitative research requires authors consider their worldview and biases^24^ therefore we sought disconfirming data to ensure we did not over-emphasize data that reinforced our existing positions. Furthermore, reflexive journaling was performed by the lead investigator and co-researchers.

### Ethics

Our Institutional Review Board approved this study [Pro00108168], and each participant gave informed consent. Participants were free to drop out of the study at anytime and without justification.

## Results

We enrolled 24 adult participants, ages ranging from the second to ninth decade of life: seven CA survivors, six family members of CA survivors and eleven family members of CA non-survivors (see Table 1 for participant descriptions). Two participants were CA survivors whose videos of their CA were available online without their consent. Nine participants were family members of CA victims whose CA was video recorded and distributed online. Thirteen participants had experience of CA that was not recorded and shared online. Interviews lasted between 29 and 74 minutes. Family relationships including spouses, unmarried partners, siblings, children, and close friends. Two husband and wife partners, one spouse and their adult daughters were interviewed as a group as well (See Table 1 for participant details). The elapsed time from CA to interview date ranged from 1 to 17 years.

**Table 1:** Participant demographic.

Participant description	N
Survivors	7
Family members of survivors	6
Family member of non-survivor	11
Video status	
Cardiac arrest video online	11
No online video of cardiac arrest	13
Family members	
Spouse	8
Daughter/son	6
Close friend	3
Participant ages	Mean (min–max)
Survivors	49 (15–83)
Family member of survivor	51.5 (28–77)
Family member of non-survivor	56 (27–69)
Years since cardiac arrest	6 (1–17)
Country of residence of participants	
Canada	10
America	7
Australia	4
United Kingdom	3

Two themes and five sub-themes were identified by our inductive analysis relating to the experience of having one’s CA video recorded and distributed (See Table 2). Five themes were also identified deductively, related to the appropriate handling and analysis of online CA videos (See Table 3). The themes identified transcended the participant’s characteristics, such as whether they were a survivor or family member or whether the CA was video recorded or not.

**Table 2:** The experience of viewing their own, or a loved-one’s, cardiac arrest video (P = Study Participant).

Core theme	Subtheme	Exemplary quotes
1. Shock, hurt and helplessness	1a. Disbelief that someone would put the video online	It was a total fucking surprise, like what the actual fuck. The worst day of our lives was stolen from us and some asshole decides it is fine to put it on Youtube? I cannot even begin to understand what would make them think this was ok. Or even what they have to gain from it. Like what’s the point, right? P16
		So there I am like, looking at the news online, right, cause Dad’s arrest was in the news. And I am reading about what the news says and the news has found a video from some guy’s Facebook that shows Dad stumbling and people crowding ‘round. I can’t believe what I’m seeing. They make his death a viral video basically and they don’t think about the effect of this on us or even care enough to let us know. P2
	1b. Reliving the old trauma and a new trauma	Watching it? Yeah, I watched it. I couldn’t not watch it. And yeah, it affected me big time. And I felt her die in my heart over and over. But it is not just the hurt of losing her anymore, it is the hurt of being betrayed by the person who has access to the security camera and the videos and who shared it. That’s what’s traumatizing now. The new trauma is it being on these websites and all the internet people, the trolls commenting about her death, how she died and making fun of it. The internet is a gross place. P18
		I might be old fashioned about this. But the human decency that is totally absent from their actions, that’s what hurt me and the family and especially my sisters. It brought back all the grief and pain of her death all over again. Like she died twice, that’s what watching it was like for me. P6
	1c. Helplessness at the permanence	It’s not like there’s even a way for us to have it taken down, right? Once it goes online it never disappears, like, whoever uploaded it pulled a trigger and the bullet cannot be taken back. You know, it is permanent and we tried so hard to find copies and have them taken down, but eventually we learned this would be totally impossible. We were totally helpless and totally made victims all over again. It is an insult-to-injury situation. P16
		It is still there you know. We have asked to have it taken down. We wrote YouTube and Google and messaged and commented on the video and maybe one gets taken down, but it just shows up again on another profile. There’s nothing we can do. P11
2. Surreality and reality	2a. Transcendence	You know, I don’t remember anything about my cardiac arrest. I guess that’s probably pretty normal. I don’t remember the entire day before it until about a full week after. So for me, my experience is like I’m watching myself, having my out of body experience through the CCTV you know? It is so weird seeing yourself die. Watching my video is like having my out of body death experience. P7
		This might sound crazy, but watching Dad have a cardiac arrest on the ice like that really puts me there with him. It is like I feel it too, along with him. A part of me dies with him there and watching him go through that, from collapse to being taken away by the paramedics, I watch it and it is like I am there, like I am transported to the arena and I am there with him. P24
	2b. Understanding the reality	I am glad I watched it. I had lots of unanswered questions, like, there was a lot of mystery around how she died and what the staff there did. It didn’t look like he suffered and that may change my mind, if she had suffered, but she didn’t look like it at all, and so I’m glad I watched it. I saw the staff and I saw mum. She had the seizure and they all stood around and she was gasping and laying still and gasping, doing the agonal breathing, and the staff were just standing around and she died there. P11
		The video shows the off duty nurse checking on him and he started CPR right away. And the policeman comes with an AED and you can see just how hard she’s trying to save his life. It is incredible, they are such heroes. P16

### Experience of having one’s CA video recorded and distributed

#### Core theme 1: Shock, hurt and helplessness

The experience of finding an online CA video was perceived as negative. Our participants reported emotional “shock” upon discovery, and being emotional “hurt” by those who uploaded the video. They also reported “helplessness” and “lack of control.”

#### Subtheme 1a: Disbelief that someone would put the video online

To further describe the experience of finding a family member’s video, participants used adjectives such as: “unbelievable,” “surprising,” “disappointing,” “incomprehensible,” and “selfish.” We interpreted this within a theme called “shock, hurt and helplessness.” Family members and survivors provided narrative explanations for why the video was made and how it came to be uploaded.

*Example of participant feedback:*

*“So there I am like, looking at the news online, right, ‘cause Dad’s arrest (CA) was in the news. And I am reading about what the news says, and the news has found a video from some guy’s Facebook that shows Dad stumbling and people crowding ‘round. I can’t believe what I’m seeing. They make his death a viral video basically, and they don’t think about the effect of this on us or even care enough to let us know.” Study Participant P 2*

#### Subtheme 1b: Reliving old emotional trauma and experiencing new emotional trauma

Watching the video (often repeatedly) was reported as reinforcing the initial traumatic experience and inflicting new trauma, as what was unwitnessed became witnessed. Notably, this was worse for family member participants than for CA survivors. The act of uploading, commenting, sharing and liking videos was described as “gross,” “lacking human decency,” “vindictive,” and “attacking.” A common theme was that the arrest (CA) was the first trauma, whereas a second trauma resulted from “betrayal,” “public humiliation,” and/or “voyeurism.”.

*Example of participant feedback:*

*Watching it? Yeah, I watched it. I couldn’t not watch it. And yeah, it affected me big time. And I felt her die in my heart over and over. But it is not just the hurt of losing her anymore, it is the hurt of being betrayed by the person who has access to the security camera and the videos and who shared it. That’s what’s traumatizing now. The new trauma is ‘it’ being on these websites and all the internet people, the trolls commenting about her death, how she died and making fun of it. The internet is a gross place.P1*

#### Subtheme 1c: Helplessness at the permanence

Our participants also reported distress regarding their lack of control over the video, specifically its possession, distribution, and permanence. Participants believed that all video hosting sites should respond to requests to stop hosting and sharing sensitive content such as CA videos when approached by survivors or families.

*Example of participant feedback:*

*“It is still there you know. We have asked to have it taken down. We wrote YouTube and Google and messaged and commented on the video and maybe one gets taken down, but it just shows up again on another profile. There’s nothing we can do”. P11*

### Core Theme 2: Surreality and Reality

For some participants, the videos were akin to a transcendent experience; survivors and family members felt “transported back to the event” and “re-immersed.” For family members, it triggered a closeness and co-experience with the victim. For both parties, despite the negative emotions, the videos also enabled sense-making and closure because they expanded their understanding of the details of the CA event. For some participants the videos provided information, they filled a knowledge gap and helped them understand what happened.

#### Subtheme 2a: Transcendence

Many participants (five of seven) also described an “out-of-body experience” from watching their own CA video. Both survivors and family members put themselves back into the event and describe the events in a disconnected and objective manner, akin to being a dispassionate narrator.

*Example of participant feedback:*

*“You know, I don’t remember anything about my (CA). I guess that’s probably pretty normal. I don’t remember the entire day before it, or until about a full week after. So for me, my experience is like I’m watching myself, having my out of body experience through the CCTV, you know? It is so weird seeing yourself die. Watching my video is like having my out of body death experience”. P7*

#### Subtheme 2b: Understanding the reality

Despite reporting negative emotions such as of distress, re-lived trauma, and helplessness, participants also reported near universal gratitude or perceived benefit after watching their CA video, and regardless of whether survivors or family members. To paraphrase, this made the event real and believable. It filled in uncertainties and solved mysteries.

*Example of participant feedback:*

*“I am glad I watched it. I had lots of unanswered questions, like, there was a lot of mystery around how she died and what the staff did. It didn’t look like she suffered. I saw the staff and I saw mum. She had the seizure and they all stood around and she was gasping and laying still and gasping, doing the agonal breathing, and the staff were just standing around and she died there. P11*

### Core Theme 3: Considerations for Educators and Researchers

Study participants advise that videos be used for education or research after ensuring their use is justified by helping future victims, considering protections for privacy of the person in the video, obtaining permission whenever possible, acting against harm, and protecting the vulnerable. Of note, participant recommendations align with established Western biomedical ethical principles (respect for autonomy, non-maleficence, beneficence and justice) which are discussed below.

#### Subtheme 3a: Proceed only when the balance of benefits outweighs the harms

Study participants emphasized the importance of using CA video recordings of real CAs only when the potential benefits outweigh the potential harms. Examples of benefits included improved rescuer education and knowledge that might help save future CA victims. Potential harms include emotional distress, reliving the experience, and privacy violation.

*Example of participant feedback:*

*“Don’t do the research just for the sake of doing research. There’s got to be some kind of justifiable benefit to victims and their families”. P8*

The recommendation is to proceed when the benefits outweigh the risks. This recommendation reflects the principles of beneficence (promote wellness) and non-maleficence (avoid causing harm); decision-making based on the balance of these principles is a common ethical pursuit in professional clinical practice.

#### Subtheme 3b: We deserve privacy

Study participants emphasized the need for privacy.

*Example of participant feedback:*

*“My personal ethics of this is that victims should have privacy. Researchers shouldn’t share anything where the victim can be recognized.” P13*

The emphasis on privacy and consent mirrors widely accepted ethical principles. In other words, the importance of patient privacy, consent and autonomy are central tenets of research, clinical medicine and nursing.

#### Subtheme 3c: Obtain permission

Study participants recommend that educators and researchers make reasonable effort to contact the CA survivor and family to obtain permission to use the video.

*Example of participant feedback:*

*“It should be the victim or their family’s decision if their video gets shared or used for research or CPR teaching, especially if you can see them. If the research only views the video but doesn’t share it, that seems a lot better to me.” P14*

Obtaining consent for the use of and sharing of identifiable videos was a paramount concern for participants and addresses survivor and family autonomy to make decisions about issues affecting them. Notably, permission was seen as less critical when videos were not further distributed or where individuals were unidentifiable or anonymous.

#### Subtheme 3d: Do not harm us

To study participants, acting in any way that could harm survivors and families was unacceptable. Furthermore, participants spoke of a duty for educators and researchers to stop the sharing of videos that were hurtful or harmful for survivors and families.

*Example of participant feedback:*

*“I think if anyone, researchers included, comes across something that is terrible. Like a video of a death, violence, hateful kind of stuff it should be reported and taken down. Researchers should be reporting things like this too.” P5*

Participants articulated that researchers and healthcare professionals have a duty to report or flag videos that could be harmful or contravene web and social media platforms’ terms of service. That is, study participants stressed the importance that professionals intervene when videos pose risks of harming others, which speaks to the ethical principle of non-maleficence.

#### Subtheme 3e: Protect the vulnerable

Participants highlighted that certain groups would be more likely to experience video surveillance and suffer a health emergency such as CA (e.g. those living in public spaces such as the streets or prisons). In other words, these individuals would likely be the subjects of a disproportionate percentage of CA videos, meaning an extreme lack of privacy and exposure of their social vulnerability.

*Example of participant feedback:*

*“I would worry that lots of these sorts of videos would come from people dying in public or in areas under lots of cameras like homeless people, or prisoners or drug users.” P22*

In this research context, the ethical principle of justice highlights the importance of fair and equitable treatment for all. It urges careful consideration of the sources of video data and the potential harms and privacy concerns, particularly for vulnarable communities.

## Discussion

Our study is the first to describe the experience of finding one’s own or a family member’s CA video online without consent. Furthermore, this is the first study to engage survivors and co-survivors in describing how educators and researchers should engage with online CA videos. Through thematic analysis of interview data, we identified experience-based themes such as shock, hurt and helplessness, and surreality and reality. Themes regarding ethical considerations to guide the use of online CA videos include: the research must help, victims deserve privacy, obtaining permission, not causing harm, and protecting the vulnerable.

The findings of this study build on the current body of knowledge related to CA, publicly recorded and shared videos, education, and research. The experience of CA, including survivorship, co-survivorship and bereavement, are associated with complex psychological sequalae^23^ and unmet care needs.^24^, ^25^ The death of a loved one is life’s most acutely impactful and long-term life events for most people.^26^, ^27^ Survivors experience depression, anxiety and post-traumatic stress disorder at higher rates than the general public.^28^ Co-survivors and caregivers also experience significant health burdens and reductions in quality of life.^29^, ^30^ The mental health effects of having a video available online without permission are unknown; however, our findings suggest they may be significant, are worthy of additional exploration and resuscitation educators and scientists must not act in a way that could harm survivors and families.

Video is a valuable tool for medical education.^31^, ^32^, ^33^ Video recordings of resuscitation have been used for over two decades to assess team performance^34^, ^35^ and more recently by bystanders to allow emergency call-takers to assess lay-responder chest compression quality.^36^, ^37^ The capture and use of video in these contexts is highly regulated; they consider the need for patient privacy and follow health system privacy and research ethics board requirements. Cardiac arrest videos captured by the public and uploaded to the internet follow no such requirements, yet have noteworthy social, forensic, ethical and possibly legal implications, much like bystander videos of violent events, and public disasters.^38^, ^39^, ^40^ The possibility of improving resuscitation education and advancing resuscitation must be balanced with the potential harm of contributing to the proliferation and sharing of online CA videos.

Cardiac arrest videos, including those captured and shared without consent, are a valuable data source for improving CA recognition and response,^41^ including training machine learning detection methods,^42^ developing curricula and improving cardiac arrest care. The online videos are a data source that requires thoughtful collection and analysis that is sensitive to the perspectives and needs affected by their existence. Future research should be undertaken but be informed by themes identified in our study (Table 3), until more rigorous guidelines are developed. Future studies of CA videos should include large sample analyses of pre- and intra-arrest victim, bystander, and rescue behaviour.

**Table 3:** Core Theme 3: Considerations for Educators and Researchers (P = Study Participant).

Subthemes	Ethical principle(s)	Exemplary quotes
3a. Proceed only when the balance of benefits outweighs the harms	Beneficence and Non-maleficence	The staff will never think it is just a seizure again I bet. The video is so powerful and I know the staff have seen it too. They did not respond because they were not very well trained. And the video shows what not to do. So, yeah, I know videos like his can help people. P11
		The video is such a valuable education tool. I’m a healthcare professional, I have my CPR certification, I have for years, and I didn’t know that cardiac arrest could look like that. I wouldn’t have known to do CPR on a person who is still breathing. I would have waited for the agonal breathing to stop, which is totally wrong. Learning from a video like K’s is really powerful. And I think we should learn from this; it is like a silver lining to his tragedy. P16
		Don’t do the research just for the sake of doing research. There’s got to be some kind of justifiable benefit to victims and their families. P8
3b. We deserve privacy	Autonomy	My personal ethics of this is that victims should have privacy. Researchers shouldn’t share anything where the victim can be recognized. P13
		And if they [the videos] are online, anyone who shares, likes, comments on and makes them more accessible or discoverable is in the wrong. These sorts of things should be confidential because they’re sacred moments and the victims and their families deserve privacy. P15
3c. Obtain permission	Autonomy	Try to get consent to use the videos whenever feasible. The family should consent or the person if they lived. This is so important if the video can identify the people involved, like the people trying to help too. P22
		It should be the victim or their family’s decision if their video gets shared or used for research or CPR teaching, especially if you can see them. If the research only views the video but doesn’t share it, that seems a lot better to me. P14
		If the research is invisible, like the researchers watch them without sharing and they view and take their measurement and their results are all summaries and anonymous description, I would feel that was acceptable. If you studied her arrest video but didn’t share it and your results did not identify her, I wouldn’t mind. You shouldn’t have to find me and get permission for that. P10
3d. Do not harm us	Non-maleficence	I think if anyone, researchers included, comes across something that is terrible. Like a video of a death, violence, hateful kind of stuff it should be reported and taken down. Researchers should be reporting things like this too. P5
		Don’t interact with videos in ways that increase their proliferation or makes them go viral. P5
		Lots of video sharing websites have terms of service right? Well, the researchers need to follow those I think. P4
3e. Protect the vulnerable	Justice	I would worry that lots of these sorts of videos would come from people dying in public or in areas under lots of cameras like homeless people, or prisoners or drug users. P22
		If the most CCTV cameras and the most people are in China, then most videos would come from there, right? This seems unfair to them. P16

This study has noteworthy limitations. Our participants are limited to Canadians, Americans and Europeans – our findings may not be generalizable to other locations. People from Eastern cultures may have important and different values regarding privacy, autonomy, justice, and the acceptability of online CA video use. We were unable to ascertain differences in participant experience based on survivor/family member demographics, time since arrest, arrest setting or participant relationship to CA victims. Our study focused on themes present across the interviews, possibly at the expense of nuanced differences between participants. Furthermore, our participants had pre-existing interests in research and improving CA education. We acknowledge our desire to use CA videos for education and research and the potential bias this may introduce; however, we employed multiple strategies to address this (disconfirming data, reflexive journaling, involvement of survivors and co-survivors as co-researchers).

Overall, according to our participants, educators and researchers should ask the following questions when using online videos of CA:

1) Will its use benefit future victims? Or is this voyeurism?
2) The privacy of the persons involved be protected? If yes, make a reasonable effort to protect the person’s identity.
3) Has the person/people in the video consented to its use? Can permission/consent be obtained? If yes, make a reasonable effort to do so.
4) Protect victims and the public from harm, report harmful content, and do not interact with harmful content in ways that promote it (views, likes impressions, saves, sharing etc.)
5) Do not use videos from specifically vulnerable populations like prisoners, homeless persons, drug users etc., without specific reasons to do so and specific considerations.

## Conclusions

Our data suggests that viewing one’s own or a family member’s online CA video can be traumatizing but may also help the viewer’s understanding of the event. Our participants acknowledged the utility and potential benefits of using CA videos for education and research but emphasized the potential harm that may result from careless online interaction with them. When using online CA videos educators and researchers have a duty to adhere to the principles of privacy, victim and family autonomy, justice, and non-malfeasance. Before undertaking any work with online CA videos potential harms and benefits must considered and reconciled.

## Funding

Alberta Registered Nurses Education Trust.

## Declaration of Competing Interest

The authors declare that they have no known competing financial interests or personal relationships that could have appeared to influence the work reported in this paper.
